# A probiotic mix partially protects against castration-induced bone loss in male mice

**DOI:** 10.1530/JOE-21-0408

**Published:** 2022-05-30

**Authors:** Lina Lawenius, Hannah Colldén, Karin Horkeby, Jianyao Wu, Louise Grahnemo, Liesbeth Vandenput, Claes Ohlsson, Klara Sjögren

**Affiliations:** 1 Sahlgrenska Osteoporosis Centre and Centre for Bone and Arthritis Research, Department of Internal Medicine and Clinical Nutrition, Institute of Medicine, University of Gothenburg, Gothenburg, Sweden; 2 Mary MacKillop Institute for Health Research, Australian Catholic University, Melbourne, Australia

**Keywords:** osteoporosis, bone mass, probiotic, gut microbiome, short-chain fatty acids

## Abstract

Studies in postmenopausal women and ovariectomized mice show that the probiotic mix *Lacticaseibacillus paracasei* DSM13434, *Lactiplantibacillus plantarum* DSM 15312 and DSM 15313 (*L.* Mix) can protect from bone loss caused by sex steroid deficiency. Whether probiotic bacteria can protect bone also in sex steroid-deficient males is less studied. We used the orchiectomized mouse as a model for age-dependent bone loss caused by decreasing sex hormone levels in males. We treated 10-week-old male mice with either vehicle (veh) or *L.* Mix for 6 weeks, starting 2 weeks before orchiectomy (orx) or sham surgery. Importantly, mice treated with *L*. Mix had a general increase in total body bone mineral density (BMD) and lean mass (*P* ≤ 0.05) compared with veh-treated mice. Detailed computer tomography analysis of dissected bones showed increased trabecular BMD of the distal metaphyseal region of the femur in *L*. Mix compared to veh-treated orx mice (+8.0%, *P* ≤ 0.05). In the vertebra, *L*. Mix treatment increased trabecular bone volume fraction BV/TV (+11.5%, *P* ≤ 0.05) compared to veh in orx mice. Also, *L*. Mix increased the levels of short-chain fatty acids (SCFAs) such as propionate and acetate and important intermediates in SCFA synthesis such as succinate and lactate in the cecal content of male mice. In conclusion, *L*. Mix treatment resulted in a general increase in BMD in adult male mice and prevented trabecular bone loss in femur and vertebra of orx mice. These bone protective effects of *L*. Mix were associated with increased levels of SCFAs in the cecal content of male mice.

## Introduction

Osteoporosis is a debilitating disease affecting one in two women and one in five men after 50 years of age (Sambrook & Cooper 2006). Both men and women lose bone in an age-dependent manner because of decreasing sex hormone levels. In women, the rapid bone loss occurring during and early after menopause contributes to the lower bone mass and higher risk of fractures in women compared with men. Probiotic bacteria can alter the composition of the gut microbiota or its metabolic activity and thereby confer a health benefit to its host (Bron *et al.* 2011). We have previously demonstrated that a probiotic mixture of three lactobacilli (*L*. Mix) can prevent bone loss in ovariectomized (ovx) female mice and postmenopausal women (Ohlsson *et al.* 2014, 2021, Jansson *et al.* 2019). Normal aging in males will cause a decrease in sex hormone levels and bone mass, and hypogonadism is an important risk factor for osteoporosis in men (Vanderschueren *et al.* 2014). Orchiectomy (orx) results in decreased bone mass in rodents as well as in humans (Stepan *et al.* 1989, Erben *et al.* 2000). Several studies have demonstrated beneficial effects of probiotics on bone mass also in male mice under various conditions, but to our knowledge, no study has investigated the effects of probiotics on bone in castrated male mice (McCabe *et al.* 2013, McCabe & Parameswaran 2018, Collins *et al.* 2019, Schepper *et al.* 2019*a*,* b*).

Probiotics can affect bone mass regulation through several possible mechanisms. Gut microbiota fermentation of dietary fiber produces short-chain fatty acids (SCFAs) that strengthens the gut barrier. SCFAs function as an energy source for intestinal epithelial cells, regulate host immune responses, promote anti-inflammatory regulatory T (Treg) cells and affect enteroendocrine cells that play an important role in host physiology by secreting hormones (Arpaia *et al.* 2013, Martin-Gallausiaux *et al.* 2021). Estrogen-deficient mice treated with SCFAs are protected against bone loss (Lucas *et al.* 2018). Not only in the liver but also in other tissues, androgens are conjugated and thereby deactivated by glucuronidation, which increases the water solubility of the compounds (Barbier & Belanger 2008). The glucuronidated androgens are then excreted in urine or via bile to the small intestine. We have earlier demonstrated that the gut microbiota is a major regulator of androgen metabolism in intestinal contents, but the effect of treatment with probiotics on androgen metabolism in the gut is unknown (Collden *et al.* 2019).

In the present study, we evaluate the bone protective effect of *L*. Mix in orx mice as a model for osteoporosis in men.

## Materials and methods

### Mouse model and treatment

C57BL/6J male mice purchased from Charles River were housed in a standard animal facility under controlled temperature (22°C), photoperiod (12 h light:12 h darkness cycle), with free access to fresh water and pellet diet (Teklad diet 2016, Envigo). Teklad diet 2016 is a fixed formula diet containing 16% protein from plant sources. Teklad diet 2016 does not contain alfalfa or soybean meal, thus minimizing the occurrence of natural phytoestrogens that can interfere in studies of castration-induced bone loss. We made the decision to use the C57BL/6J mouse strain since we used this strain in earlier studies of the effect of probiotic bacteria on castration-induced bone loss and wanted to be able to compare the results (Ohlsson *et al.* 2014, 2021, Lawenius *et al.* 2020, 2022). At 12 weeks of age, the mice were randomized into four groups (*n* = 13–15) and subjected to orx or sham surgery under inhalation anesthesia with isoflurane (Baxter Medical AB, Kista, Sweden). The number of mice in each group was decided based on experience from earlier studies with similar treatments and parameters studied (Ohlsson *et al.* 2014, 2021, Lawenius *et al.* 2020, 2022). In these studies, we found small but consistent effects on the bone with the number of observations ranging from 8 to 15 per group. Mice were treated with either *Lacticaseibacillus* *paracasei* DSM13434, *Lactiplantibacillus* *plantarum* DSM 15312 and DSM 15313 (*L.* Mix) or vehicle (veh, maltodextrin) for 6 weeks starting 2 weeks before orx or sham surgery. The freeze-dried probiotic bacteria were given in the drinking water at a concentration of 10^9^ colony-forming units (cfu)/mL and water bottles were changed daily. Vigorous shaking for 1–2 min dispersed the bacteria in the water. The survival of the bacteria in the water bottles was checked regularly, and after 24 h, the concentration dropped to 50%. The probiotic bacteria were provided by Probi AB (Lund, Sweden). At the end of the study, mice were anesthetized with Ketador/Dexdomitor (Richter Pharma/Orion Pharma), bled from the axillary vein, and thereafter killed by cervical dislocation. Blood was allowed to coagulate for at least 30 min at room temperature and centrifuged for 10 min, the serum was then collected and frozen. Tissues for RNA preparation and cecal contents were snap-frozen in liquid nitrogen. Bones were excised and fixed in 4% paraformaldehyde. The treatment allocation was blinded for the scientist doing the analyses but not for the scientist handling the mice. All experimental procedures involving animals were approved by the regional animal ethics committee in Gothenburg (ethics number 19/467).

### Dual-energy X-ray absorptiometry (DXA)

Total body bone mineral density (BMD), fat percentage, and lean mass were analyzed using Faxitron UltraFocus dual-energy X-ray absorptiometry (DXA) (Faxitron Bioptics, Tuscon, AZ, USA). Mice were scanned using a X-ray energy of 40 kV and 0.28 mA for 2.53 s with a spatial resolution of 24 µm using 2× geometric magnification. Images were analyzed using the software VISION DXA software (Faxitron Bioptics).

### Peripheral quantitative computed tomography (pQCT)

Peripheral quantitative CT (pQCT; XCT Research m, Stratec Medizintechnik GmbH, Germany) was used to analyze the trabecular and cortical compartments of the femur, operating at a resolution of 70 µm (Windahl *et al.* 1999). Briefly, trabecular bone was analyzed in the metaphyseal region of the femur. The scan was positioned in the metaphysis at a distance corresponding to 3% of the total length of the femur from the distal growth plate and the trabecular bone region was defined by setting an inner area to 45% of the total cross-sectional area. The cortical bone was analyzed in the mid-diaphyseal region at 36% of the total length of the femur from the distal growth plate.

### High-resolution microCT (µCT)

High-resolution µCT analyses were performed using Skyscan 1172 scanner (Bruker MicroCT, Aartselaar, Belgium) as previously described (Moverare-Skrtic *et al.* 2014). Briefly, the vertebra (L5) was imaged with an X-ray tube voltage of 50 kV, a current of 200 µA and a 0.5 mm aluminum filter. The scanning angular rotation was 180° and the angular increment was 0.70°. The voxel size was 6.54 µm isotropically. NRecon (version 1.6.9) was used to perform the reconstruction after the scans. The trabecular bone in the vertebral body caudal of the pedicles was selected for analysis within a conforming volume of interest (cortical bone excluded) commencing at a distance of 5 µm caudal of the lower end of the pedicles and extending a further longitudinal distance of 230 µm in the caudal direction.

### Gene expression analysis

RNA from vertebral bodies (L3 and L6) was extracted using TriZol Reagent (Sigma) followed by RNeasy Mini QIAcube Kit (Qiagen). Real-time PCR analyses were run using StepOnePlus Real-Time PCR systems (Applied Biosystems). Predesigned probes for *Rankl* (*Tnfsf11*, Mm00441908_m1), *Opg* (*Tnfrsf11b*, Mm00435452_m1), *Il-6* (Mm00446190_m1), *Runx2* (Mm00501580_m1), *ColI* *αI* (Mm00801666_g1), *Trap* (*Acp5*, Mm00475698_m1), *Ctsk* (Mm00484036_m1) and *Tnf* *α* (Mm00443258_m1) were used (Applied Biosystems). The mRNA abundance of each gene was calculated using the ΔΔCt method, adjusted for expression of 18S rRNA (4310893E, Applied Biosystems) and presented as relative expression.

### Flow cytometry

Bone marrow cells from one femur were isolated and erythrocytes were lysed using 0.83% ammonium chloride. Cells were stained with eBioscience™ Fixable Viability Dye eFluor™ 780 according to the manufacturer’s protocol (Invitrogen, ThermoFisher Scientific). Cells were extracellularly stained with anti-CD3-BV510 (Clone 17A2, Nordic BioSite AB, Täby, Sweden), anti-CD4-FITC (Clone RM4-5, Nordic BioSite AB) and anti-CD25-APC (Clone 3C7, BD, Franklin Lakes, NJ, USA). Cells were then fixed and permeabilized using the FoxP3 staining buffer kit (Invitrogen, Thermofisher Scientific) and intracellularly stained with anti-Foxp3-PE (Clone FJK-16s, ThermoFisher Scientific) according to the manufacturer’s instruction. Treg cells were defined as CD4+CD25+Foxp3+, and results are expressed as the frequency of live cells. Samples were run on BD FACSVerse (BD), and data were analyzed using FlowJo software (version 10.4.1).

### Short-chain fatty acids

Cecal SCFAs were measured using gas chromatography coupled to mass spectrometry detection (GC-MS) as described previously (Wichmann *et al.* 2013). Briefly, approximately 20–100 mg of cecal content was mixed with internal standards, added to glass vials and freeze-dried. All samples were then acidified with HCl, and SCFAs were extracted with two rounds of diethyl ether extraction. The organic supernatant was collected, the derivatization agent N-tert-butyldimethylsilyl-N-methyltrifluoroacetamide (Sigma-Aldrich) was added and samples were incubated at room temperature overnight. SCFAs were quantified with a gas chromatograph (Agilent Technologies 7890A) coupled with a mass spectrometer (Agilent Technologies 5975C). SCFA standards were obtained from Sigma-Aldrich.

### Androgen analysis

Steroids were extracted, derivatized and measured as described previously (Nilsson *et al.* 2015, Collden *et al.* 2019). Briefly, cecal contents were weighed, added to PBS (PBS) and homogenized by shaking with a 5 mm steel bead in a Tissuelyzer II for 5 min. Serum samples were measured volumetrically by pipetting and diluted with PBS. After the addition of isotope-labeled standards, sex steroids were extracted by liquid–liquid extraction, followed by solid-phase extraction. Derivatization was performed in two steps: oximation followed by esterification. Testosterone and dihydrotestosterone (DHT) were separated with gas chromatography and detected with electron capture negative chemical ionization by an Agilent 7000 triple quadrupole mass spectrometer (Agilent). All peaks were integrated using the MassHunter quantitative analysis workstation software from Agilent. The measured concentration was corrected for the amount of input material (wet mass of intestinal contents or volume of serum). In serum, the lower level of quantification (LLOQ) was 8 pg/mL for testosterone and 2.5 pg/mL for DHT (Nilsson *et al.* 2015), and in cecum, the LLOQ was 40 pg/g for testosterone and 20 pg/g for DHT (Collden *et al.* 2019). Values below the LLOQ were set to half of the LLOQ in order not to overestimate undetectable hormone levels.

### Serum analyses

Enzyme immunoassay kits were used to measure the bone resorption marker collagen type I C-terminal telopeptides (CTX-1, Immunodiagnostics Systems, Herlev, Denmark) and the bone formation marker procollagen type I N-terminal propeptide (PINP, Immunodiagnostics Systems, Herlev, Denmark) in serum according to the manufacturer’s directives.

### Statistical analyses

GraphPad Prism was used for all statistical analyses. Results are presented as means ± s.e.m. Our main analysis is the two-way ANOVA where we calculated the overall effect of treatment (Veh/*L*. Mix), surgical procedure (sham/orx) and their interaction. We then did a* post hoc* analysis to calculate the effect of *L*. Mix compared to veh treatment in sham and orx mice, respectively, using Sidak’s* post hoc* test corrected for multiple comparisons. If we had an overall effect of *L*. Mix vs veh or an interaction effect in the two-way ANOVA, we performed the* post hoc* test to see if the effect was mainly in the sham or the orx group or in both. When only two groups were compared, between-group differences were calculated using the two-tailed Student’s* t-*test. *P* ≤ 0.05 was considered significant. Normal distribution was analyzed using the Shapiro–Wilk test. If the sample distribution did not pass the normality test, values were normalized by log transformation before analysis.

## Results

### *L.* Mix treatment increased total body BMD and lean mass in male mice

**Figure 1:**
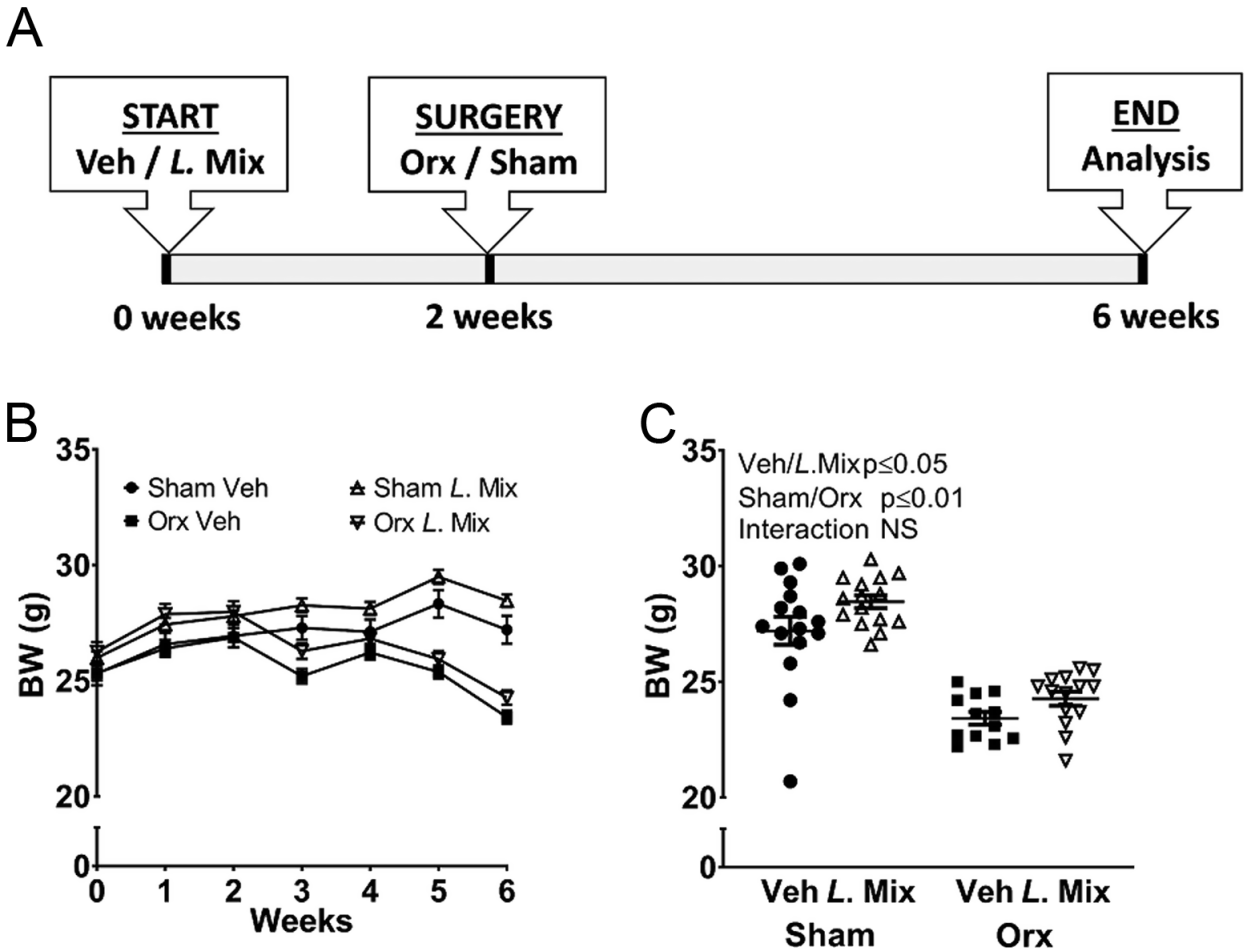
Treatment of adult mice with a mixture of three lactobacilli strains resulted in an increase in body weight. Ten-week-old male mice were treated with either vehicle (veh) or a mixture of three lactobacilli (*L*. Mix) for 6 weeks, starting 2 weeks before orchiectomy (orx) or sham surgery. Study protocol (A). Body weight (BW) curve (B). Values are given as mean ± s.e.m. (*n* = 13–15). Body weight at the end of study (C). Symbols in the scatter plots represent individual mice and the bars indicate mean ± s.e.m. The overall effects of treatment (Veh/*L*. Mix), surgical procedure (sham/orx) and their interaction were calculated using two-way ANOVA, NS, not significant.

**Figure 2:**
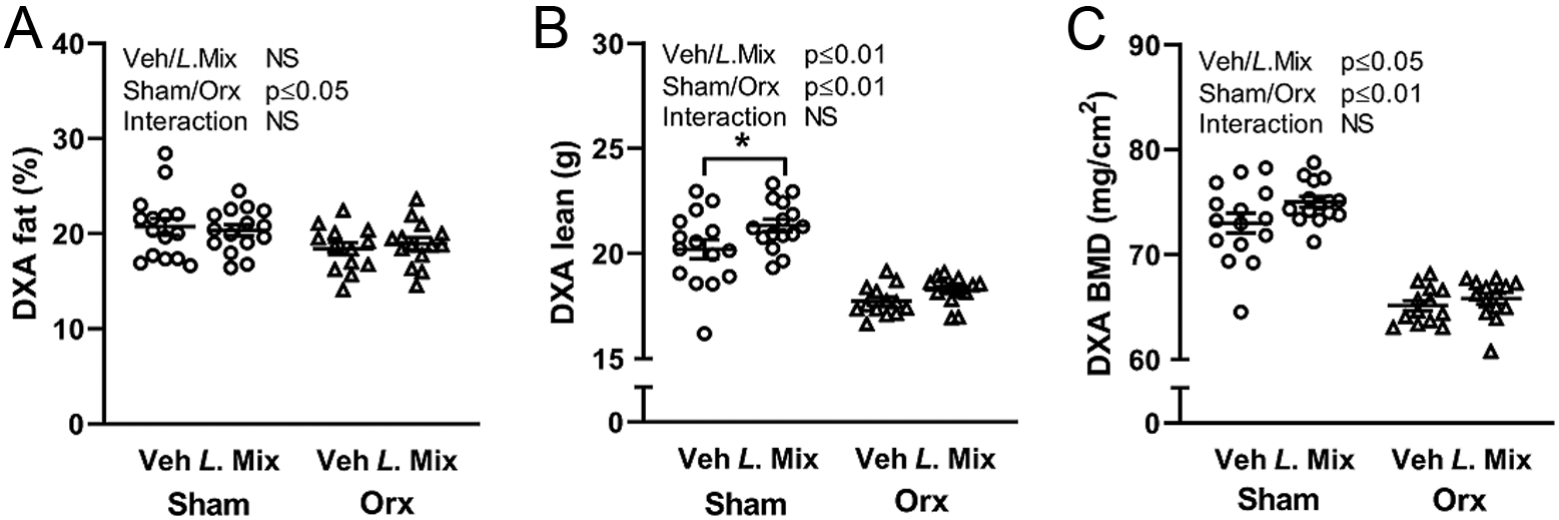
*L.* Mix treatment increased total body BMD and lean mass in male mice. Ten-week-old male mice were treated with either vehicle (veh) or a mixture of three lactobacilli (*L*. Mix) for 6 weeks, starting 2 weeks before orchiectomy (orx) or sham surgery. At the end of the experiment, mice were analyzed with whole-body DXA to measure (A) percentage of fat, (B) lean mass and (C) areal bone mineral density (BMD) of whole body. Symbols in the scatter plots represent individual mice and the bars indicate mean ± s.e.m. (*n* = 13–15). The overall effects of treatment (Veh/*L*. Mix), surgical procedure (sham/orx) and their interaction were calculated using two-way ANOVA, NS, not significant. The effect of *L*. Mix compared to veh treatment in sham and orx mice, respectively, was calculated by Sidak’s* post hoc* test to correct for multiple comparisons, *P ≤ 0.05.

We treated 10-week-old male mice with either veh or a mixture of three probiotic bacteria, *Lacticaseibacillus paracasei* DSM 13434, *Lactiplantibacillus plantarum* DSM 15312 and DSM 15313 (*L.* Mix) for 6 weeks, starting 2 weeks before orx or sham surgery (Fig. 1A). As expected, orx mice gained less body weight compared to sham mice (Fig. 1B and C). Interestingly, treatment with *L*. Mix resulted in a general increase in body weight (Fig. 1B and C). Body composition analysis with DXA and organ dissection at the end of the study demonstrated that orx mice have decreased fat percent and weight of gonadal fat compared to sham mice, but *L.* Mix treatment had no effect on these parameters (Fig. 2A and Supplementary Table 1, see section on supplementary materials given at the end of this article). Lean mass was decreased with orx, but treatment with *L.* Mix resulted in a general increase in lean mass (Fig. 2B). A substantial part of lean mass is muscle tissue, but the relative weights of the dissected muscles quadriceps and levator ani were not affected by *L.* Mix treatment ( Supplementary Table 1). As expected, orx mice had a decrease in whole-body BMD (Fig. 2C). Importantly, mice treated with *L*. Mix had a general increase in whole-body BMD (Fig. 2C).

### *L.* Mix treatment increased trabecular bone mass in orx mice

**Figure 3:**
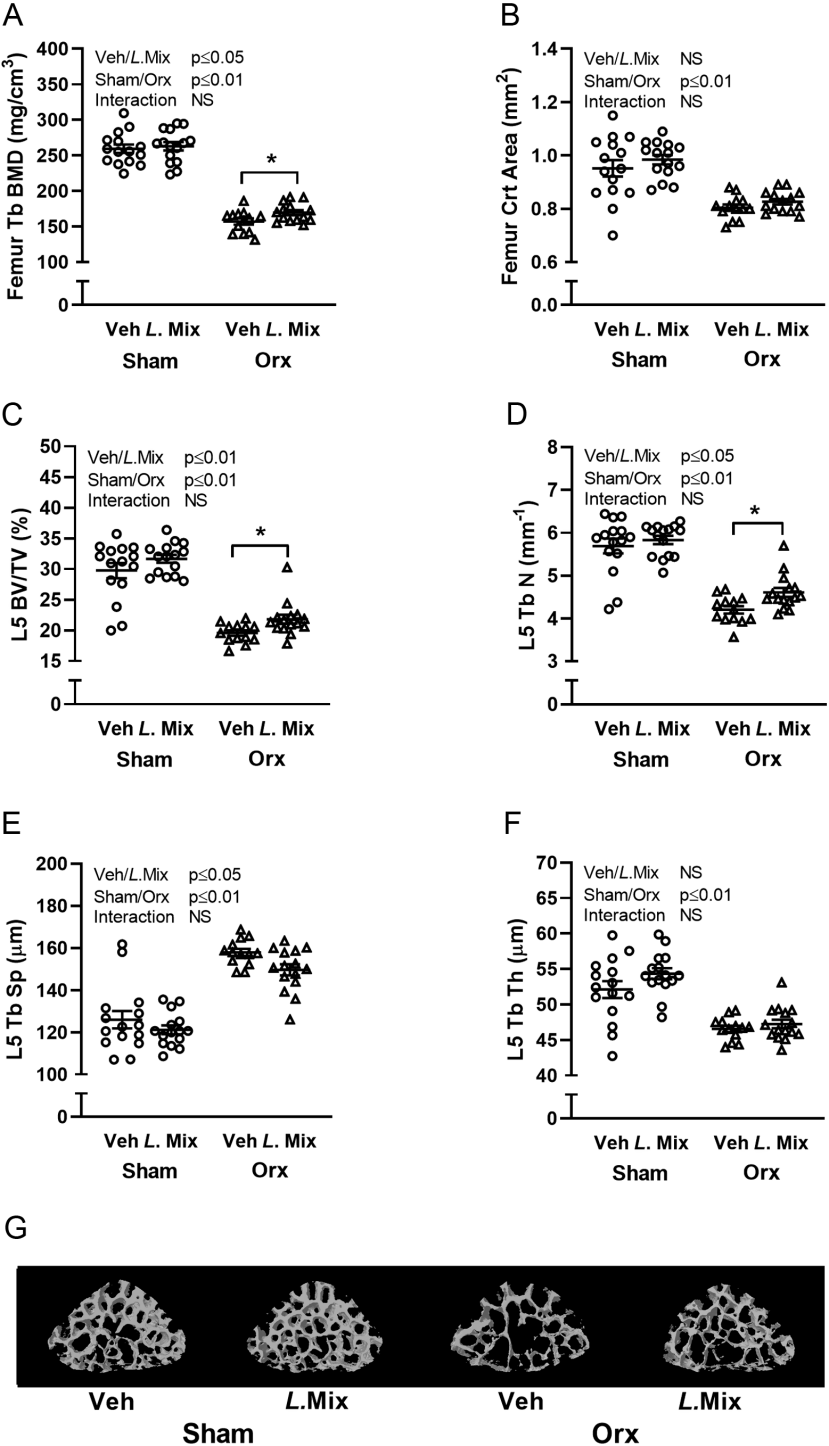
*L.* Mix treatment increased trabecular bone mass in orx mice. Ten-week-old male mice were treated with either vehicle (veh) or a mixture of three lactobacilli (*L*. Mix) for 6 weeks, starting 2 weeks before orchiectomy (orx) or sham surgery. At the end of the experiment, dissected femurs were analyzed with peripheral quantitative CT (pQCT) to measure (A) trabecular bone mineral density (Tb BMD) and (B) cortical area (Crt area). Dissected vertebral body (L5) were analyzed with high-resolution μCT to measure (C) trabecular bone volume fraction (BV/TV), (D) trabecular thickness (Tb Th), (E) trabecular separation (Tb Sp) and (F) trabecular number (Tb N). Representative μCT images of L5 with cortical bone were excluded (G). Symbols in the scatter plots represent individual mice and the bars indicate mean ± s.e.m. (*n* = 13–15). The overall effects of treatment (Veh/*L*.Mix), surgical procedure (sham/orx) and their interaction were calculated using two-way ANOVA, NS, not significant. The effect of *L*. Mix compared to veh treatment in sham and orx mice respectively was calculated by Sidak’s* post hoc* test to correct for multiple comparisons, **P ≤ 0.01 and *P ≤ 0.05.

**Figure 4:**
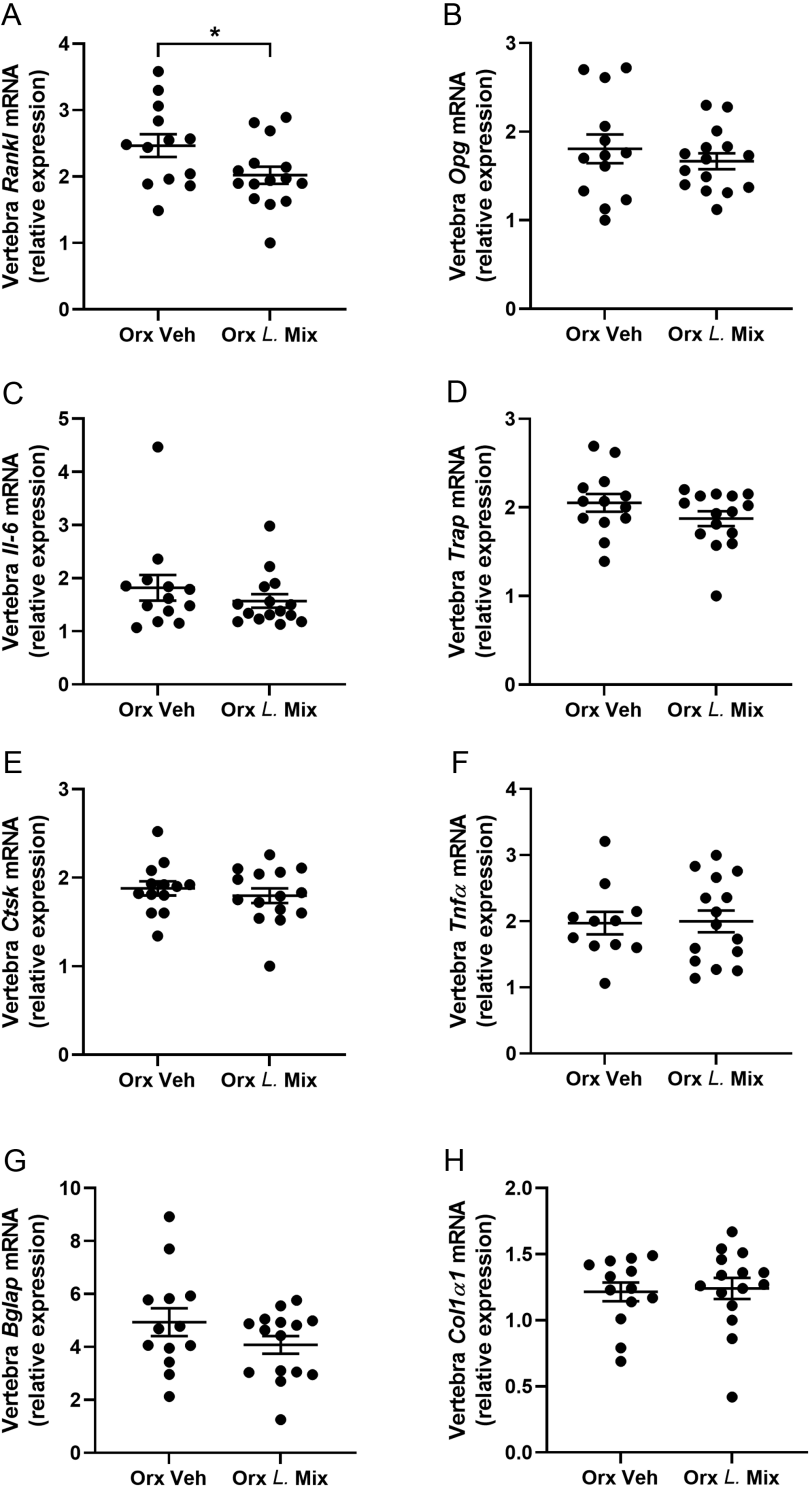
Gene expression in the vertebra of orx mice after treatment with *L.* Mix. Ten-week-old male mice were treated with either vehicle (veh) or a mixture of three lactobacilli (*L*. Mix) for 6 weeks, starting 2 weeks before orchiectomy (orx) or sham surgery. Real-time PCR analysis of gene expression known to affect bone turnover in the vertebra (L3 and L6): receptor activator of nuclear factor-κB ligand (*Rankl*, A), osteoprotegerin (*Opg*, B), interleukin 6 (*Il-6*, C), tartrate resistant acid phosphatase (*Trap*, D), cathepsin K (*Ctsk*, E), tumor necrosis factor α (*Tnfα*, F), bone gamma-carboxyglutamate protein (*Bglap*, G) and Collagen, type Iα1 (*Col1α1*, H). Symbols in the scatter plots represent individual mice and the bars indicate mean ± s.e.m. (*n* = 13–15). *P ≤ 0.05 vs the orx veh group. Between-group differences were calculated using Student’s* t-*test.

Detailed CT analysis of dissected bones showed increased trabecular BMD of the femur in *L*. Mix compared to veh-treated orx mice but no effect in cortical bone (Fig. 3A and B). In the vertebra, *L*. Mix treatment increased trabecular bone volume fraction (BV/TV) and trabecular number compared to veh treatment in orx mice (Fig. 3C, D and G). Treatment with *L*. Mix also resulted in a general decrease in trabecular separation in the vertebra (Fig. 3E and G). The effect of the *L*. Mix vs veh treatment expressed in percent, both in the Orx and veh group, for the main bone parameters is presented in Supplementary Table 2. The Tb BMD of the femur was 8.0 ± 3.5% higher in orx mice treated with *L*. Mix compared with veh (*P* ≤ 0.05, Supplementary Table 2). In the vertebra, the BV/TV was 11.5 ± 4.5% higher in orx mice treated with *L*. Mix compared with veh (*P* ≤ 0.05, Supplementary Table 2). For the sham groups, the increase was not statistically significant indicating that although the combined effect of *L*. Mix in the sham and orx group is statistically significant in the two-way ANOVA, the effect is more pronounced in the orx than in the sham group (Supplementary Table 2). Since *L.* Mix resulted in a general increase in body weight (Fig. 1B and C) and lean mass (Fig. 2B), we corrected femur trabecular BMD and lumbar vertebra BV/TV for body weight. After correction for body weight, there is still a loss of trabecular bone in femur and vertebra with castration and only a tendency for a protective effect on trabecular bone with *L.* Mix (Supplementary Fig. 1). This indicates that *L.* Mix has an effect on the whole body including the bone. At the end of the study, we measured bone turnover markers in serum and both the formation marker PINP and the resorption marker CTX-1 were increased with orx but treatment with *L*. Mix had no effect (Supplementary Fig. 2A and B). We compared expression of genes important in bone mass regulation in the vertebra of orx mice and found that *L.* Mix treatment decreased the expression of the osteoclast promoting gene *Rankl* compared to veh treatment (Fig. 4A). However, other genes indicating osteoclast formation and activity (*Opg, Trap, Ctsk*), osteoblast formation (*Bglap, Col1α1*) and inflammation (*Il-6, Tnfα*) were not significantly changed by *L.* Mix treatment (Fig. 4B, C, D, E, F, G and H).

### *L*. Mix treatment increased the SCFAs propionate and acetate in the gut

**Figure 5:**
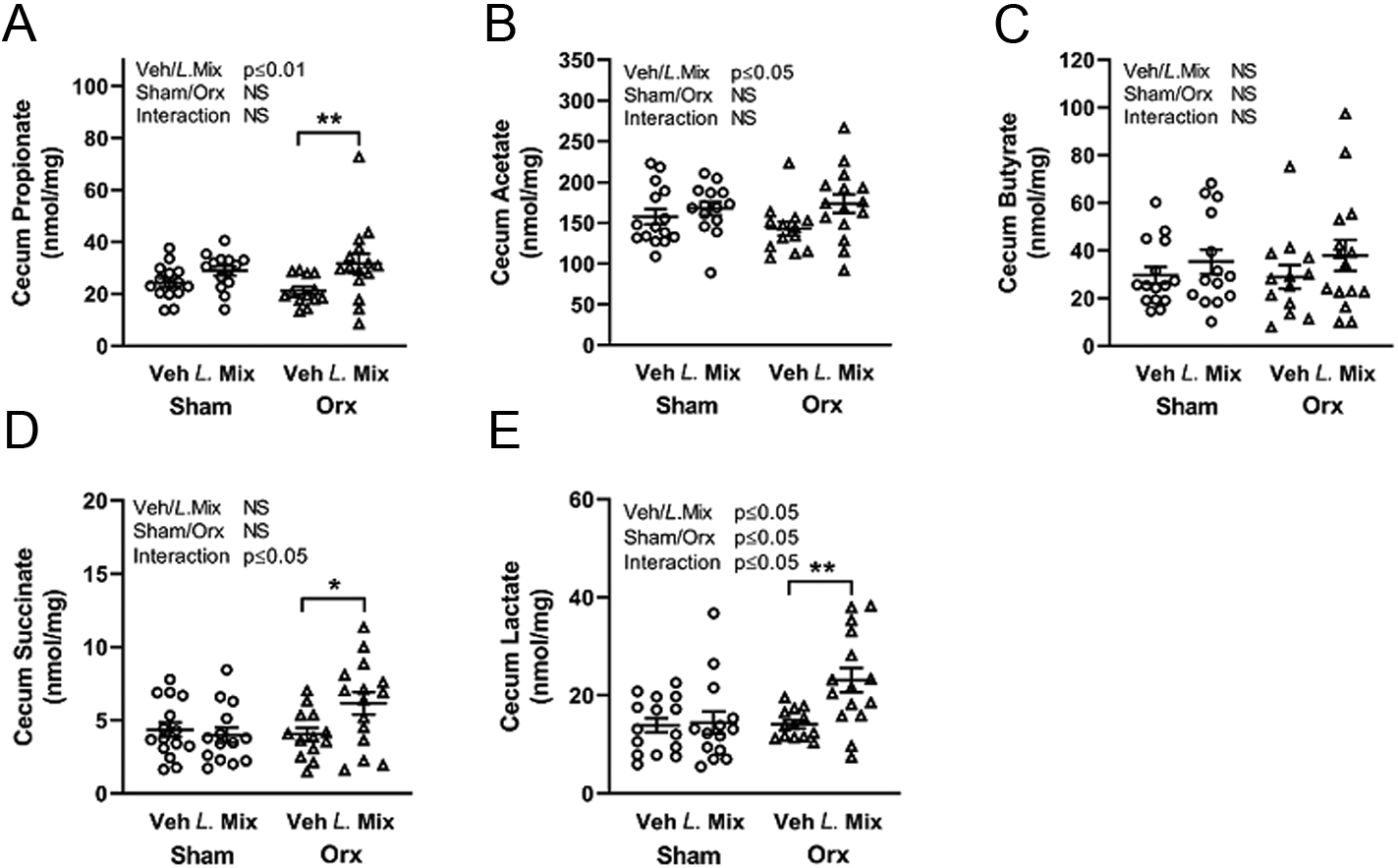
*L.* Mix treatment increased the SCFAs propionate and acetate in the gut. Ten-week-old male mice were treated with either vehicle (veh) or a mixture of three lactobacilli (*L*. Mix) for 6 weeks, starting 2 weeks before orchiectomy (orx) or sham surgery. At the end of the study, we measured cecal short-chain fatty acids (SCFAs) propionate (A), acetate (B), butyrate (C) and intermediates in SCFAs production succinate (D) and lactate (E). Symbols in the scatter plots represent individual mice and the bars indicate mean ± s.e.m. (*n* = 13–15). The overall effects of treatment (Veh/*L*. Mix), surgical procedure (sham/orx) and their interaction were calculated using two-way ANOVA, NS, not significant. The effect of *L*. Mix compared to veh treatment in sham and orx mice, respectively, was calculated by Sidak’s* post hoc* test to correct for multiple comparisons, **P ≤ 0.01 and *P ≤ 0.05.

Probiotics have the capacity to ferment dietary fiber and produce bone protective SCFAs that can be absorbed by the host (Louis & Flint 2017, Lucas *et al.* 2018, Tyagi *et al.* 2018). A 6-week treatment with *L*. Mix increased the cecal content of the SCFAs propionate and acetate compared with veh, and a similar trend (*P* = 0.16) was observed for butyrate (Fig. 5A, B and C). Two important intermediates in SCFA synthesis are succinate and lactate. *L*. Mix increased succinate in orx but not in sham mice (Fig. 5D). *L*. Mix increased lactate and, similar to succinate, this effect was more pronounced in orx mice, supported by a significant interaction factor (Fig. 5E).

### No effect of *L*. Mix treatment on sex steroid levels in serum or gut

We have earlier demonstrated that the gut microbiota is a major regulator of androgen metabolism in intestinal contents but there was no effect of *L*. Mix treatment on testosterone or DHT in neither serum nor intestinal contents of cecum (Supplementary Table 3). As expected, testosterone and DHT in serum decreased markedly following orx confirming successful castration (Supplementary Table 3).

### No effect of *L*. Mix treatment on regulatory T-cells in bone marrow of male mice

Orx decreased the frequency of Treg cells in the bone marrow of male mice, similar to what we have previously observed in ovx female mice (Supplementary Fig. 3) (Ohlsson *et al.* 2014). Treatment with *L*. Mix had no effect on the frequency of Treg in bone marrow (Supplementary Fig. 3).

## Discussion

We have previously demonstrated that *L*. Mix can prevent bone loss in ovx female mice and postmenopausal women. In the present study, we show for the first time that *L*. Mix treatment protects bone also in sex hormone-deficient male mice. We used the orx mouse as a model for male osteoporosis. We treated 10-week-old male mice with either veh or *L.* Mix for 6 weeks, starting 2 weeks before orx or sham surgery. *L.* Mix treatment resulted in a general increase of body weight, lean mass and BMD compared with veh in male mice. *L*. Mix protected trabecular bone in the femur and vertebra of orx mice but had no effect on cortical bone. The bone protective effects were associated with an increase in the SCFAs propionate and acetate and two important intermediates in SCFA synthesis, succinate and lactate, in the cecal content of male mice.

*L.* Mix. treatment resulted in a general increase in whole-body BMD in male mice, and detailed analysis showed that the effect was on the trabecular bone compartment both in the femur and the vertebra with a more pronounced effect in orx compared to sham mice. Two earlier studies showed that *Limosilactobacillus reuteri* increased the trabecular bone mass in the femur and vertebra of gonadal intact male mice (McCabe *et al.* 2013, Collins *et al.* 2019). *L. reuteri* also has beneficial effects on bone in male mice with low bone mass due to glucocorticoid treatment, diabetes type 1 or long-term antibiotic treatment (Zhang *et al.* 2015, Schepper *et al.* 2019*a*,*b*). Antiretroviral agents can trigger a bone loss in human immunodeficiency virus patients, and in male mice, *Lacticaseibacillusrhamnosus GG* attenuates bone loss induced by the antiretroviral agent tenofovir disoproxil fumarate (Liu *et al.* 2019). Together, these studies suggest that probiotic treatment may have beneficial effects on bone in male mice with various conditions of bone loss but the present study is the first demonstrating a bone protective effect in a male mouse model of low bone mass due to sex hormone deficiency. After correction for the increased body weight with *L.* Mix, there was only a tendency for a protective effect on the trabecular bone with *L.* Mix. One may speculate that the increased levels of SCFAs in the gut with *L.* Mix may have a general effect that is not specific to bone. SCFAs have several metabolic functions serving both as energy sources for the gut epithelial cells and contributing to gluconeogenesis in the liver that may cause anabolism (Louis & Flint 2017). In our earlier studies, when we treated ovariectomized female mice with *L*. Mix, we saw a protective effect on bone but no effect on body weight with *L*. Mix (Ohlsson *et al.* 2014, 2021). However, the effect of castration on body composition is completely different for male and female mice. Male mice lose weight with castration while female mice gain weight mainly as an effect of increased fat mass. To summarize, we have a protective effect of *L*. Mix on bone after castration in both female and male mice, but in male mice, this is accompanied with a general increase in body weight with *L*. Mix. In the vertebra of orx mice, *L*. Mix decreased the expression of *Rankl*, which is a major promotor of osteclastogenesis. This finding indicates that the effect of *L*. Mix may be mediated via reduced bone resorption but no effect on other osteoclastic genes or serum bone turnover markers was observed. Furthermore, the *P*-value was not corrected for multiple testing and a significance level of *P* < 0.0062 would be required if we had corrected for eight tests using the conservative Bonferroni correction method when measuring gene expression.

In the present study, we show for the first time that *L.* Mix treatment changes the metabolic capacity of the gut microbiota. *L.* Mix treatment increased the SCFAs propionate and acetate and two important intermediates in SCFAs synthesis succinate and lactate in the cecal content of male mice. An earlier study in ovx mice showed that both feeding of a high fiber diet and treatment with SCFAs can protect from bone loss due to estrogen deficiency (Lucas *et al.* 2018). According to Lucas *et al.,* SCFAs in serum had a direct effect on osteoclastogenesis (Lucas *et al.* 2018). In the present study, we found decreased expression of *Rankl* in vertebra after treatment of orx mice with *L.* Mix suggesting decreased osteoclast activation. We found that orx decreased the frequency of Treg cells in bone marrow, similar to what we previously reported for ovx (Ohlsson *et al.* 2014). There was no effect of *L.* Mix on the frequency of Treg in bone marrow although there was a tendency for an increase in *L.* Mix compared to veh-treated orx mice. SCFAs are known to interact with immune cells in the gut and they can induce Treg cells and a more anti-inflammatory environment both in the gut and in peripheral organs (Arpaia *et al.* 2013, Furusawa *et al.* 2013, Smith *et al.* 2013). In a study by Tyagi *et al.*, treatment of female mice with the SCFA butyrate increased bone mass, and the effect was dependent on the induction of Treg cells (Tyagi *et al.* 2018). In *L*. Mix-treated orx mice, the tendency of an increased frequency of Treg cells in bone marrow may contribute to the protective effect of *L*. Mix on bone but this needs further investigation.

We showed in a previous study that the gut microbiota deconjugates androgens in cecum and that conventionally raised mice have high levels of testosterone and DHT in cecum and colon compared with mice raised in a sterile environment with no bacteria in the gut (Collden *et al.* 2019). High levels of deconjugated androgens in the gut may have implications for androgen-related diseases in the distal intestine, and changing the gut microbiota composition and/or metabolism by treatment with probiotics might modulate intestinal androgen metabolism. Furthermore, in a study by Poutahidis *et al.,* the probiotic *L. reuteri*, when given to male mice from 5 until 12 months of age, could prevent age-related weight gain and increase the serum testosterone levels (Poutahidis *et al.* 2014). In the present study, treatment with *L.* Mix had no effect on the levels of testosterone or DHT in neither cecum nor serum of orx and sham mice. Compared with Poutahidis *et al.*, when other *L.* strains were used, the mice were younger and treated for a much shorter time (6 weeks).

To conclude, we show for the first time that *L*. Mix treatment protects trabecular bone in sex hormone-deficient male mice. The expression of *Rankl* was decreased in trabecular bone of orx mice indicating reduced osteoclast activation. *L.* Mix treatment changed the metabolic capacity of the gut microbiota, increasing the SCFAs propionate and acetate and two important intermediates in SCFAs synthesis succinate and lactate in the cecal content of male mice. One may speculate that *L*. Mix treatment increases bone mass via increased SCFA synthesis in male mice.

## Supplementary Material

Supplementary Material

## Declaration of interest

C O and K S are listed as inventors on a patent application regarding the impact of probiotics on bone metabolism and K S has received research funding for probiotic-related research from Probi AB. The other authors have nothing to disclose.

## Funding

This study was supported by the Swedish Research Council (grant number 2018-02589) and by grants from the Swedish state under the agreement between the Swedish government and the county councils (the ALF-agreement: 238261), the Lundberg Foundation (grant number 2017-0081), the Torsten Söderberg Foundation (grant number M65115), Probi AB and the Knut and Alice Wallenberg Foundation (grant number KAW 2015.0317).

## Author contribution statement

Study design: L L, H C, L V, C O, and K S. Study conduct: L L, H C, K S. Data collection: L L, H C, K L G, J W, L G, L V, and K S. Data analysis: L L, H C, J W, L V, C O, and K S. Data interpretation: L L, H C, L V, C O, and K S. Drafting manuscript: L L, H C, C O and K S. Revising manuscript: L L, H C, C O and K S. Approving final version of manuscript: L L, H C, K L G, J W, L G, L V, C O, and K S. L L, H C, L V, C O and K S take responsibility for the integrity of the data analysis.
