# Supplementary material for: A probiotic mix partially protects against castration-induced bone loss in male mice
Source: J Endocrinol. 2022 May 30;254(2):91–101. doi: 10.1530/JOE-21-0408 (PMC9254303; doi:10.1530/JOE-21-0408)
Supplement: Supplementary Material [file supplementary_material.pdf]

Supplemental Material

A probiotic mix partially protects against castration-induced bone loss in male mice

Lina Lawenius[1], Hannah Colldén[1], Karin L Gustafsson[1], Jianyao Wu[1], Louise Grahnemo[1], Liesbeth Vandenput[1,2], Claes Ohlsson[1] and Klara Sjögren[1]

[1]Sahlgrenska Osteoporosis Centre and Centre for Bone and Arthritis Research, Department of Internal Medicine and Clinical Nutrition, Institute of Medicine, University of Gothenburg, Gothenburg, Sweden. [2]Mary MacKillop Institute for Health Research, Australian Catholic University, Melbourne, Australia.

Supplemental Table 1. Organ weights corrected for body weight of orchiectomized mice treated with *L.* Mix

	Treatment	Surgery		2-way ANOVA (p value)		
		**Sham**	**Orx**	**Sham/Orx**	**Veh/ *L*.Mix**	**Interaction**
Liver (%BW)	**Veh**	4.01±0.07	3.56±0.07	p<0.0001	p= 0.0185	NS
	***L*.Mix**	4.10±0.06	3.81±0.07*			
M. Quadriceps (%BW)	**Veh**	0.812±0.014	0.858±0.028	p= 0.0156	NS	NS
	***L*.Mix**	0.792±0.015	0.838±0.014			
Gonadal fat (%BW)	**Veh**	1.69±0.12	1.08±0.16	p<0.0001	NS	NS
	***L*.Mix**	1.65±0.09	1.08±0.15			
Spleen (%BW)	**Veh**	0.294±0.014	0.379±0.012	p<0.0001	NS	NS
	***L*.Mix**	0.301±0.011	0.369±0.009			
Thymus (%BW)	**Veh**	0.113±0.004	0.284±0.011	p<0.0001	NS	NS
	***L*.Mix**	0.106±0.003	0.305±0.010			
M. Levator Ani (%BW)	**Veh**	0.319±0.015	0.117±0.006	p<0.0001	NS	NS
	***L*.Mix**	0.295±0.010	0.133±0.006			
Seminal Vesicles (%BW)	**Veh**	0.897±0.043	0.080±0.006	p<0.0001	NS	NS
	***L*.Mix**	0.852±0.026	0.079±0.003			

Ten-week-old male mice were treated with either vehicle (veh) or a mixture of three lactobacilli (*L*. Mix) for 6 weeks, starting 2 weeks before orchiectomy (orx) or sham surgery. At the end of the study tissues were dissected and weighed. Values are given as mean±SEM (n=13-15). The overall effects of treatment (Veh/*L*.Mix), surgical procedure (sham/Orx) and their interaction were calculated using two-way ANOVA. NS=not significant. The effect of

L.Mix compared to veh treatment in sham and orx mice respectively was calculated by Sidak's post hoc test to correct for multiple comparisons, **$p \leq 0.01$ and *$p \leq 0.05$.

Supplemental Table 2. Effect of *L*. Mix versus veh treatment for the main bone parameters affected by the probiotic treatment

	Sham		Orx	
	**% change**	**p**	**% change**	**p**
Femur Tb BMD (mg/cm^3)	1.37 (3.22)	NS	8.02(3.53)	0.0318
L5 BV/TV (%)	6.47 (4.72)	NS	11.54 (4.46)	0.0157
L5 Tb N (mm^{-1})	2.44 (3.46)	NS	9.66 (3.34)	0.0076
L5 Tb Sp (μm)	-3.77 (3.65)	NS	-5.24 (2.07)	0.0178

Data are percent change (SEM) for *L*. Mix versus Veh treatment in the sham and orx group (n=13-15). The effect of *L*.Mix compared to veh treatment in sham and orx mice respectively were calculated by two-tailed Student's *t*-test, NS=not significant.

Supplemental Table 3. Sex hormones in serum and intestinal contents of cecum of orchiectomized mice treated with *L.* Mix

	Treatment	Surgery		2-way ANOVA (p value)		
		**Sham**	**Orx**	**Sham/Orx**	**Veh/ *L.*Mix**	**Interaction**
Serum T (pg/ml)	**Veh**	2621±826	8±4	$p=0.0002$	NS	NS
	***L.*Mix**	4215±1370	5±1			
Serum DHT (pg/ml)	**Veh**	71.4±19.7	2.3±0.4	$p<0.0001$	NS	NS
	***L.*Mix**	96.1±27.9	1.9±0.0			
Cecum T (pg/g)	**Veh**	276.1±34.6	20.0±0.0	$p<0.0001$	NS	NS
	***L.*Mix**	281.5±33.6	22.1±2.1			
Cecum DHT (pg/g)	**Veh**	536.1±100.9	17.1±2.2	$p<0.0001$	NS	NS
	***L.*Mix**	563.6±90.7	16.5±3.4			

Ten-week-old male mice were treated with either vehicle (veh) or a mixture of three lactobacilli (*L*. Mix) for 6 weeks, starting 2 weeks before orchiectomy (orx) or sham surgery. At the end of the study, we measured sex hormones in serum and intestinal contents of cecum using gas chromatography-tandem mass spectrometry. Testosterone (T), Dihydrotestosterone (DHT). Values are given as mean±SEM (n=13-15). The overall effects of treatment (Veh/*L*.Mix), surgical procedure (sham/Orx) and their interaction were calculated using two-way ANOVA, NS=not significant.

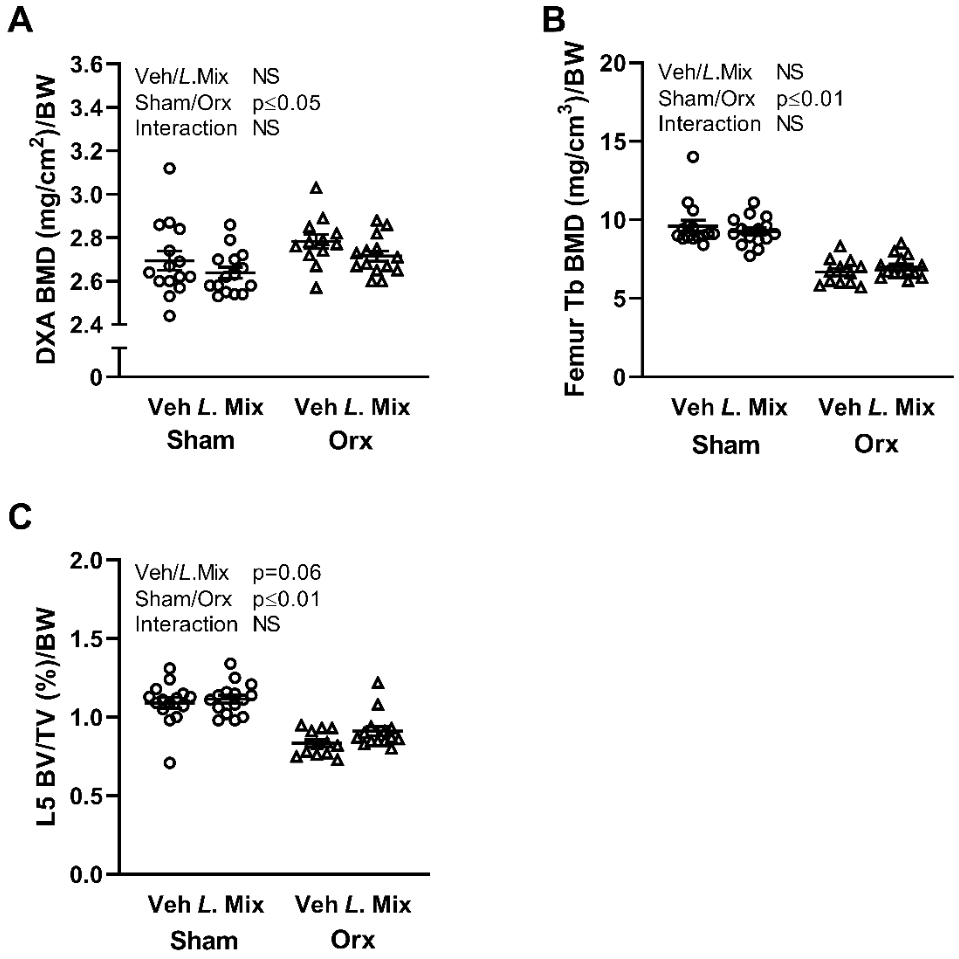

Supplemental Figure 1. Bone parameters corrected for body weight. Ten-week-old male mice were treated with either vehicle (veh) or a mixture of three lactobacilli (*L.* Mix) for 6 weeks, starting 2 weeks before orchiectomy (orx) or sham surgery. At the end of the experiment, mice were analysed with whole body DXA to measure (A) areal bone mineral density (BMD) of whole body corrected for body weight, dissected femurs were analysed with peripheral quantitative computed tomography (pQCT) to measure (B) trabecular bone mineral density (Tb BMD) corrected for body weight and dissected vertebral body (L5) were analysed with high-resolution µCT to measure (C) trabecular bone volume fraction (BV/TV) corrected for body weight. Symbols in the scatter plots represent individual mice and the bars indicate mean±SEM (n=13-15). The overall effects of treatment (Veh/*L.*Mix), surgical

procedure (sham/orx) and their interaction were calculated using two-way ANOVA, NS=not significant.

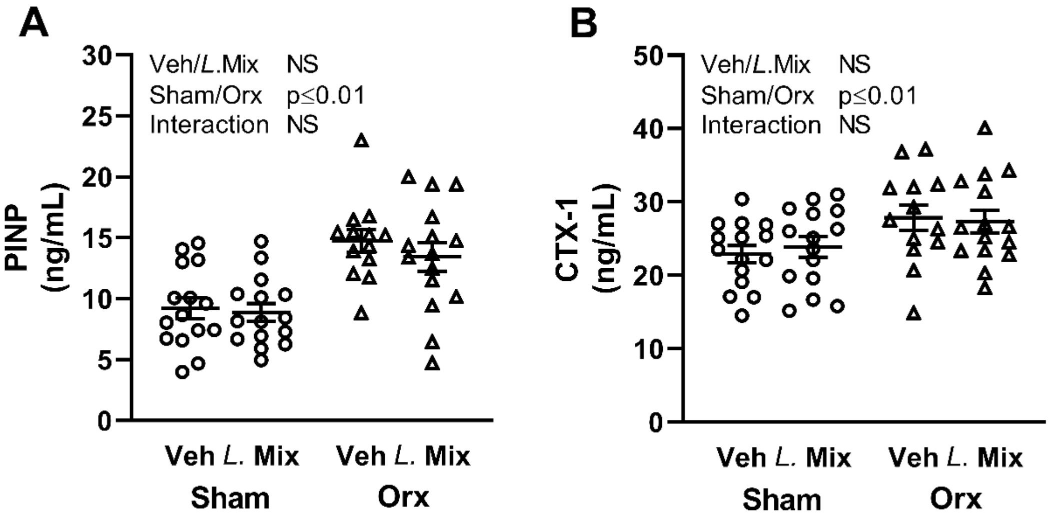

Supplemental Figure 2. *L.* Mix treatment had no effect on bone turnover markers in serum. Ten-week-old male mice were treated with either vehicle (veh) or a mixture of three lactobacilli (*L*. Mix) for 6 weeks, starting 2 weeks before orchiectomy (orx) or sham surgery. At the end of the study, we measured bone turnover markers in serum: procollagen Type 1 N-terminal propeptide (PINP, A) and C-telopeptide cross-link type 1 collagen (CTX-1, B). Symbols in the scatter plots represent individual mice and the bars indicate mean±SEM (n=13-15). The overall effects of treatment (Veh/*L*.Mix), surgical procedure (sham/Orx) and their interaction were calculated using two-way ANOVA, NS=not significant.

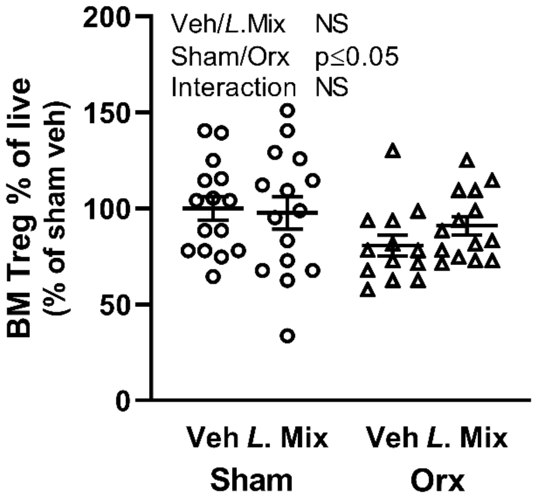

Supplemental Figure 3. Frequency of regulatory T-cells in bone marrow of mice treated with *L.* Mix

Ten-week-old male mice were treated with either vehicle (veh) or a mixture of three lactobacilli (*L.* Mix) for 6 weeks, starting 2 weeks before orchiectomy (orx) or sham surgery. At the end of the study bone marrow (BM) cells from dissected femur were stained with antibodies recognizing CD4, Foxp3 and CD25. Values represent the frequency of regulatory T cells (Treg, CD4+CD25+Foxp3+) of gated live cells. Values are presented as the percentage of the sham veh group. Symbols in the scatter plots represent individual mice and the bars indicate mean±SEM (n=11-15). The overall effects of treatment (Veh/*L.*Mix), surgical procedure (sham/Orx) and their interaction were calculated using two-way ANOVA, NS=not significant.
